# Non-neutralizing antibody responses control acute Crimean-Congo hemorrhagic fever virus infection in naïve mice

**DOI:** 10.1128/jvi.00987-26

**Published:** 2026-09-14

**Authors:** Kimberly Meade-White, Shanna S. Brown, Samantha Ertl, Natalie McCarthy, Thomas Tipih, Heinz Feldmann, David W. Hawman

**Affiliations:** 1 Laboratory of Virology, Rocky Mountain Laboratories, NIH/NIAID 549113 , Hamilton, Montana, USA; 2 Laboratory of Viral Zoonoses, Integrated Research Facility Frederick, NIH/NIAID 827085 , Frederick, Maryland, USA; University of North Carolina at Chapel Hill, Chapel Hill, North Carolina, USA

**Keywords:** mouse model, B-cells, antibody, Crimean Congo, CCHFV

## Abstract

Crimean-Congo hemorrhagic fever virus (CCHFV) is the cause of a sometimes-severe viral hemorrhagic fever, CCHF. Severe cases of CCHF often have little-to-no CCHFV-specific antibody responses prior to death. However, it is unclear if the failure to mount an antibody response is the cause of a poor outcome or a correlate of a failed host response. Here, we used a mouse-adapted strain of CCHFV (MA-CCHFV) to investigate the requirement of humoral immunity in control of acute CCHFV infection in immunocompetent mice. We found that mice infected with MA-CCHFV develop a rapid non-neutralizing antibody response, consistent with humans infected with CCHFV. Further, we found that the absence of humoral immunity in male but not female mice resulted in near-uniform mortality. Lastly, we determined that complement but not activating Fc-receptors nor the cytoplasmic Fc-receptor tripartite motif containing protein 21 contributed to antibody-mediated control of MA-CCHFV infection. Together, our findings identify rapid humoral immune responses as a critical component of the host response to acute CCHFV infection in naive hosts.

**IMPORTANCE:**

Crimean-Congo hemorrhagic fever virus (CCHFV) is the cause of a serious viral hemorrhagic fever in humans. Severe disease and poor outcomes are correlated with little-to-no CCHFV-specific antibody. However, it is unknown if the lack of CCHFV-specific antibody is a direct cause or merely a correlate of a poor outcome. Here, we investigated the contribution of humoral immunity in control of acute CCHFV infection in naive immunocompetent mice. We found that mice develop a rapid, non-neutralizing antibody response that is crucial for control of the acute infection. Further, we found that loss of complement but not activating Fc receptors or the cytoplasmic Fc receptor TRIM21 worsened disease. Together, our findings suggest that lack of CCHFV-specific antibody responses may directly contribute to poor outcomes in CCHFV-infected humans.

## INTRODUCTION

Crimean-Congo hemorrhagic fever virus (CCHFV) is a tick-borne virus in the *Bunyaviricetes* class that causes Crimean-Congo hemorrhagic fever (CCHF) (1). CCHF is a viral hemorrhagic fever (VHF), and like other VHFs, such as those caused by Ebola virus, the disease starts as a nonspecific febrile illness that can rapidly progress to hemorrhagic manifestations (1, 2). CCHFV is spread by the bite of ticks, mainly *Hyalomma* species ticks (3), or exposure to infected livestock (1). Human-to-human spread is possible, typically during care of infected patients (1, 2). In endemic areas, contact with ticks and livestock is frequent, and thus, the initial exposure may go unnoticed, and recognition of the early symptoms of CCHF is difficult (1). Therefore, CCHF patients will present to health care systems often exhibiting advanced disease when treatments like ribavirin will have limited effect (4). Currently, there are no licensed vaccines for CCHFV; thus, treatment is typically limited to supportive care.

The host determinants of CCHF outcomes are poorly understood. CCHF appears to be a uniquely human disease as serological evidence of CCHFV infection has been reported in a wide variety of species without overt disease (5). In humans, disease can vary from asymptomatic to severe, lethal disease (1), but it is unclear what mechanisms determine the disease outcome. Risk factors for poor outcomes are associated with a dysregulated inflammatory response (6, 7), uncontrolled viral replication (8–11), liver damage (12), and little-to-no CCHFV-specific antibody response (13–15). However, it is unclear whether the failure to mount an antibody response leads to poor outcomes or whether it is a symptom of a failed or pathologic host response to the infection. If antibodies are required, whether they exert control through classical neutralization or Fc-effector functions is unclear. Vaccine studies have identified a key role for non-neutralizing antibodies against the CCHFV nucleoprotein (16, 17), while neutralizing antibodies against the viral Gc or non-neutralizing antibodies against the GP38 accessory protein have prophylactic and therapeutic effects (18–21). We previously developed a mouse-adapted strain of CCHFV and showed in Rag1 knock-out mice (Rag1^−/−^), deficient in all adaptive immunity, that adaptive immunity is required to survive the infection (22). We further identified a requirement for CD8 T-cells and IFNγ (23). However, depletion of both CD4 and CD8 T-cells together did not fully recapitulate the phenotype of Rag1^−/−^ mice, suggesting a role for humoral immunity in control of the infection. Here, we used this model to evaluate the contribution of B-cells and antibody in control of acute CCHFV infection in naïve mice.

## RESULTS

### Adaptive immunity is required for survival in MA-CCHFV-infected mice

We have previously shown that adaptive immunity is required for survival in MA-CCHFV-infected mice (22). Here, we further investigated if a lack of adaptive immunity leads to uncontrolled viral replication. We found that MA-CCHFV-infected mice deficient in adaptive immunity (Rag1^−/−^) had increased weight loss, and all mice succumbed to the infection (Fig. 1a), consistent with our previous studies. We then evaluated viral RNA loads in the blood, liver, and spleen, key tissues for CCHFV replication. At day 6 post-infection (PI), shortly before terminal disease in Rag1^−/−^ mice, viral RNA loads in the blood, liver, and spleen were significantly increased in female Rag1^−/−^ mice compared to respective WT mice (Fig. 1b). Viral RNA loads were significantly increased in the blood of male mice and trended higher in the liver and spleen (Fig. 1b). Together, these data show that adaptive immunity quickly exerts control of the infection within 6 days of infection, and in the absence of these adaptive immune responses, uncontrolled viral replication likely leads to terminal disease.

**Fig 1:**
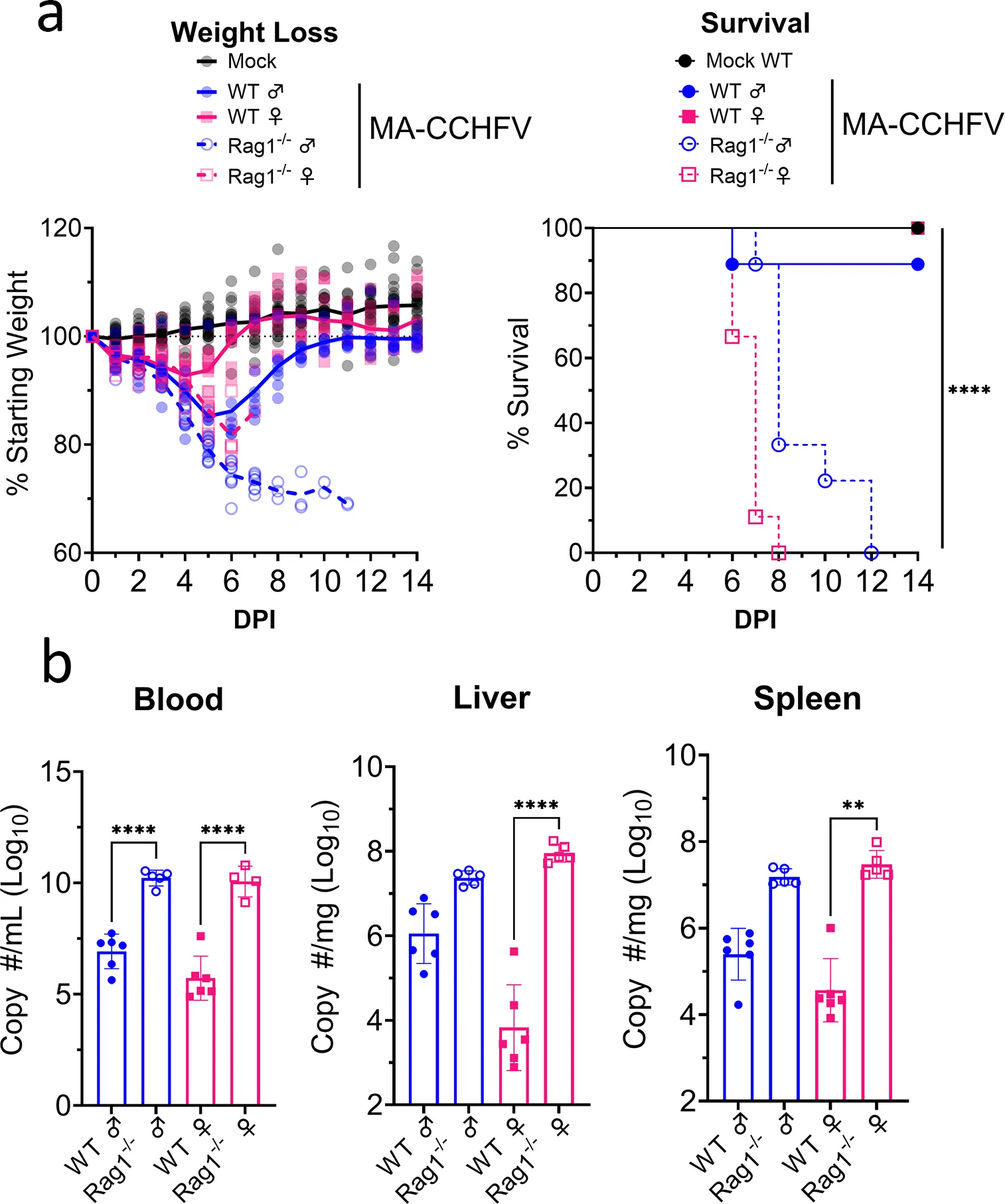
Mice deficient in adaptive immunity succumb to MA-CCHFV infection. (**a**) Groups of WT or Rag1^−/−^ mice were infected with MA-CCHFV and weighed daily and monitored for survival. *N* = 23 per group (mock) or 9 per group. *P* values calculated with a log-rank test with Bonferroni’s correction for multiple comparisons. *P* values < 0.0125 were considered significant. (**b**) Viral loads in the blood, liver, and spleen at day 6 PI were quantified by qRT-PCR. *P* values calculated with the Kruskal-Wallis test with Dunn’s multiple comparisons test. **P < 0.01; ****P < 0.0001. *N* = 5–6 per group.

### MA-CCHFV-infected mice develop rapid but non-neutralizing antibody responses

We next evaluated the humoral immune response to CCHFV in MA-CCHFV-infected mice. By whole-virion enzyme-linked immunosorbent assay (ELISA), both male and female mice developed a CCHFV-specific IgM response by day 6 PI, which declined thereafter (Fig. 2a). CCHFV-specific IgG was detectable by day 6 PI and peaked by day 21 PI (Fig. 2a), demonstrating the IgG response to CCHFV is also rapid. Using recombinant proteins, we next evaluated which viral proteins this CCHFV-specific IgG response targeted. At day 8 PI, compared to mock-infected mice, we measured significant responses against the CCHFV NP and Gc, which further increased in magnitude by day 28 PI (Fig. 2b). The overall magnitude of the response against Gc was greater than the response against NP, indicating that Gc is the dominant target of humoral immunity in MA-CCHFV-infected mice. Interestingly, despite human survivors of CCHFV developing antibodies against the CCHFV GP38 (24), responses against the CCHFV GP38 protein in MA-CCHFV-infected mice at day 8 or day 28 PI were marginal (Fig. 2b). Together, these data suggest that MA-CCHFV-infected mice develop rapid antibody responses against the CCHFV NP and Gc.

**Fig 2:**
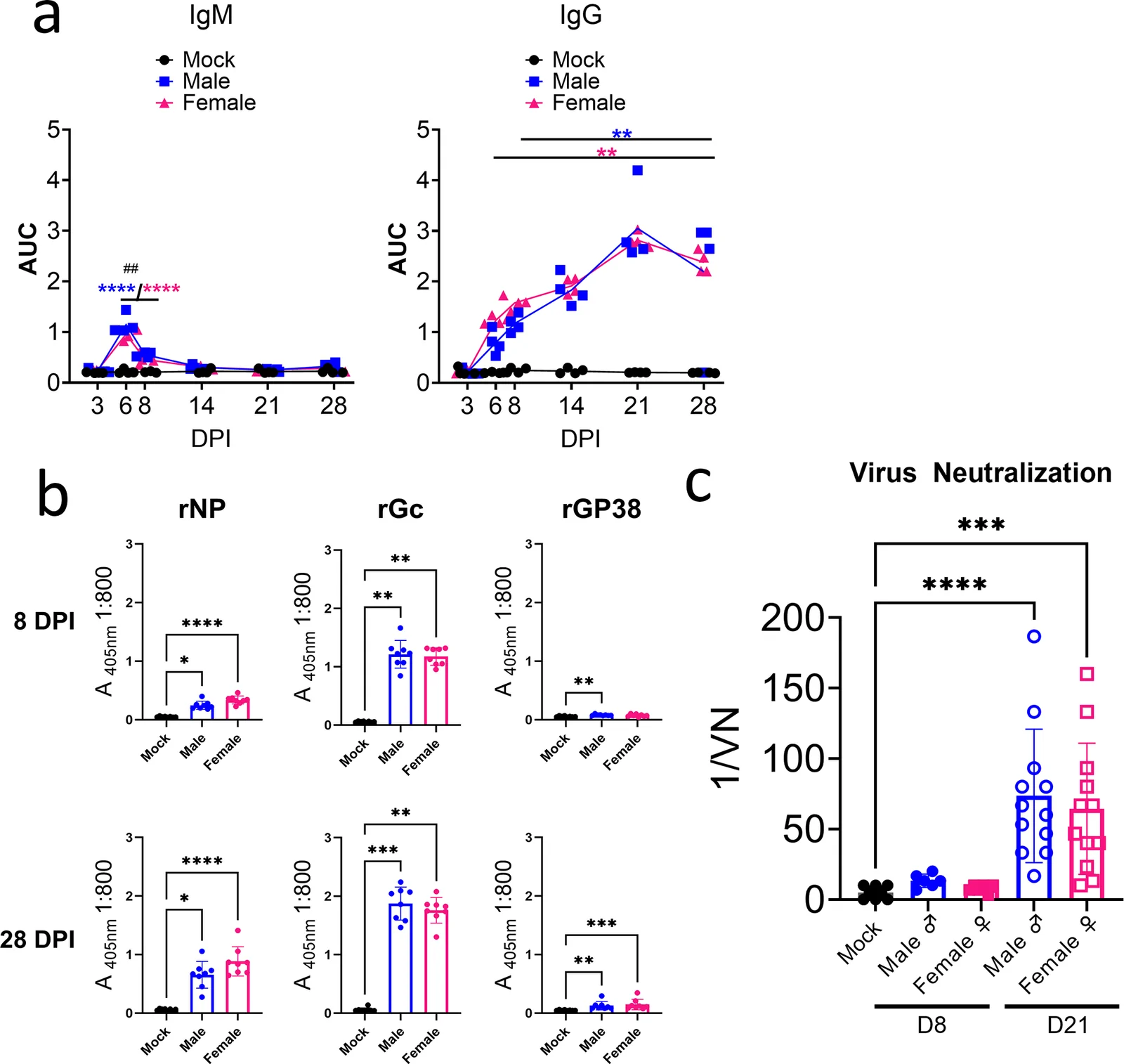
MA-CCHFV-infected mice develop a rapid, non-neutralizing CCHFV-specific antibody response. (**a**) WT mice were infected with MA-CCHFV, and CCHFV-specific IgM or IgG was measured by whole-virion ELISA. *P* values were calculated with a two-way analysis of variance (ANOVA) and Tukey’s multiple comparisons test. *N* = 4 per group. (**b**) IgG against specific viral proteins was measured at a single 1:800 dilution of serum using recombinant viral proteins at day 8 or day 28 PI. *P* values were calculated with a nonparametric Kruskal-Wallis test as diagnostics for residuals and homoscedasticity showed significantly different standard deviations between groups and non-normal distribution of residuals. *N* = 8 per group. (**c**) Serum neutralizing activity was evaluated with a microneutralization assay against authentic CCHFV. *P* values were calculated with Kruskal-Wallis test with Dunn’s multiple comparisons test. *N* = 6–12 per group. *P < 0.05, **P < 0.01, ***P < 0.001, and ****P < 0.0001. (**a**) ** and **** indicate significance of comparisons between infected male or female mice and mock-infected mice. ## *P* < 0.01 for comparison between male and female infected mice.

As Gc is the target of neutralizing antibodies (25, 26), we evaluated whether this Gc-specific response could neutralize infectious CCHFV. Although we measured CCHFV Gc-specific IgG by day 8 PI, we did not measure neutralizing activity in the serum (Fig. 2c). However, by day 21 PI, we detected significantly increased neutralizing activity compared to serum from mock-infected mice (Fig. 2c). As mice are beginning to recover by day 8 (Fig. 1a), despite no detectable neutralizing activity at this time point, this suggests that neutralizing antibody responses are not necessary for control of the acute infection in MA-CCHFV-infected mice.

### Humoral immunity is required for survival in MA-CCHFV-infected mice

We next evaluated whether this rapid CCHFV-specific antibody response was important for control of the infection. We infected WT or mice deficient in mature B-cells (μMT) with MA-CCHFV and monitored mice for clinical disease. Male μMT mice had worse disease, as evidenced by increased weight loss compared to their respective WT controls, and nearly all male μMT mice succumbed to the infection (Fig. 3a), suggesting humoral immunity to CCHFV is critical for survival in male mice. In contrast, female μMT mice had only slightly increased weight loss, and survival was not impacted, with 100% of mice surviving (Fig. 3a). Notably, female μMT mice had less weight loss than even WT male mice. These data indicate that even in the complete absence of humoral immunity, female mice retain better control of the infection than WT male mice. At day 6 PI, both male and female μMT mice had significantly increased viral loads in the blood, while female μMT mice also had significantly increased viral loads in the liver (Fig. 3b). Viral loads trended higher in the livers of male µMT mice and in spleens of both male and female µMT mice, but these differences were not significant. The similar viral loads in the livers of male WT and μMT mice (Fig. 3b) are in contrast to our previous report showing increased viral loads of the livers of male T-cell-depleted mice (23). This suggests that cellular immunity may be the primary adaptive immune response that controls the infection in the liver of CCHFV-infected male mice. As expected, infected μMT did not develop any CCHFV-specific Ig response (Fig. 3c).

**Fig 3:**
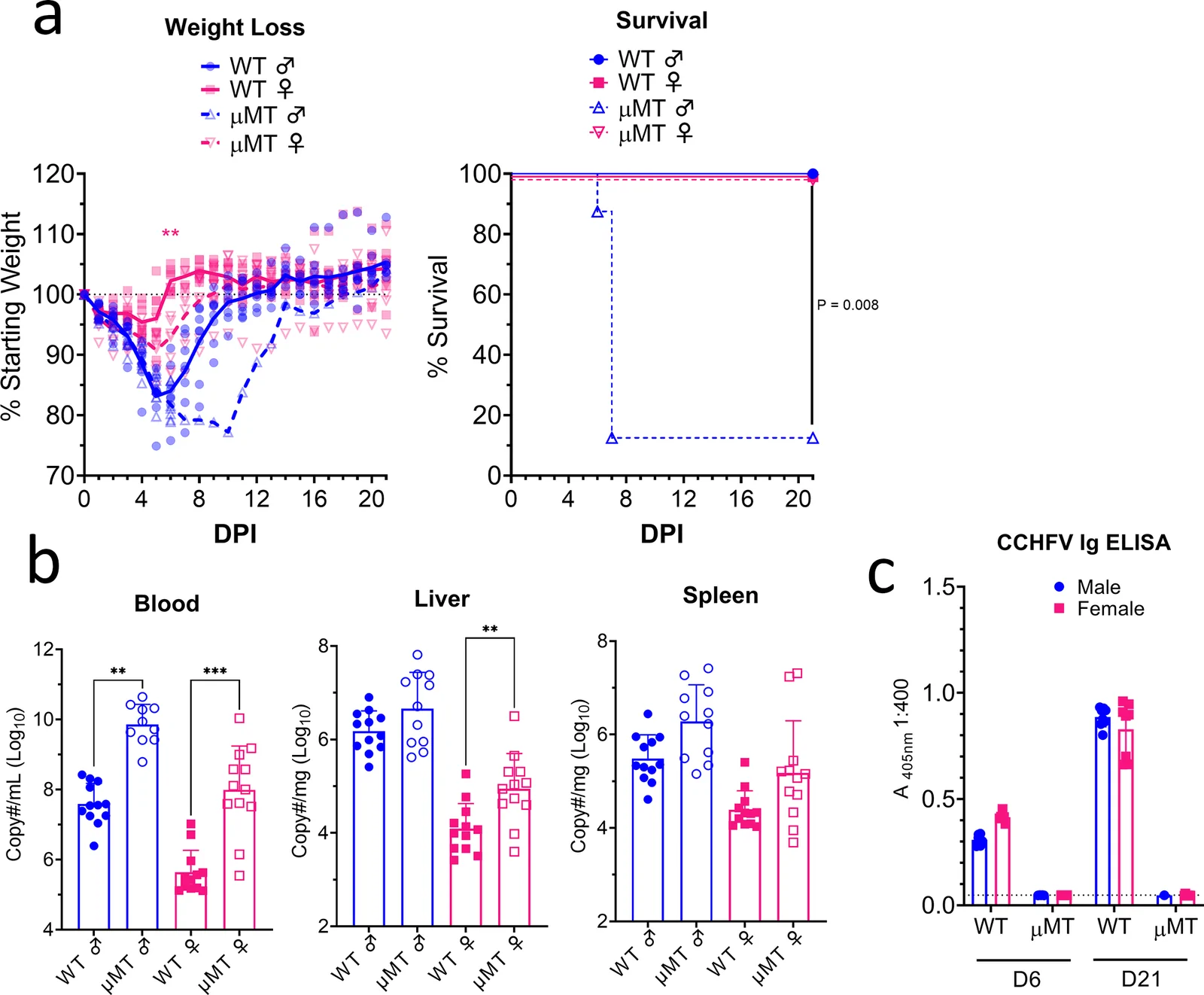
B-cells are required for control of acute CCHFV infection. (**a**) WT or B-cell-deficient μMT mice were infected with MA-CCHFV and weighed daily and monitored for survival.(**b**) Viral RNA in the blood, liver, and spleen measured by qRT-PCR. (**c**) CCHFV-specific antibody was measured by whole-virion ELISA using secondary antibody to detect any antibody isotype. *P* values calculated using a mixed-effect analysis with Tukey’s multiple comparisons test or log-rank test with Bonferroni correction (**a**). *P* values were calculated using the Kruskal-Wallis test with Dunn’s multiple comparisons test due to non-gaussian residuals (blood and spleen) or an ordinary one-way ANOVA with Sidak’s multiple comparisons test (liver). *P < 0.05, **P < 0.01, ***P < 0.001, and ****P < 0.0001. *N* = 10–12 per group (**c**). Absence of serum antibody was confirmed by whole virus ELISA and anti-Ig detection antibodies. *N* = 1 (male µMT at day 21), while the rest were 5–8 per group.

Due to the complete lack of B-cells in μMT mice, lymphoid architecture can be disrupted, leading to impaired cellular immunity (27). In our system, this could lead to defects in both humoral and cellular immunity and confound our results. To exclude this explanation, we infected MD4 transgenic mice, which contain a knock-in B-cell receptor specific for an irrelevant antigen. This results in mice with normal levels of B-cells but nearly all B-cells being specific for this irrelevant antigen (28). Similar to B-cell-deficient μMT mice, MD4 mice had worsened disease, and all male MD4 mice succumbed to the infection (Fig. S1a and b). Intriguingly, female MD4 mice had increased disease, and one female MD4 mouse succumbed to the infection (Fig. S1a and b). Unexpectedly, female MD4 mice also developed CCHFV-specific IgG by day 21, albeit reduced from WT survivors (Fig. S1d). These data indicate slightly worsened disease in female MD4 mice compared to B-cell-deficient mice, which correlated with a diminished but not absent antibody response.

### IgM or IgG can prevent death in MA-CCHFV-infected mice

At day 6 PI, when WT mice begin to recover, MA-CCHFV-infected WT mice had both CCHFV-specific IgM and IgG (Fig. 2a); thus, it was unclear which response was required to control the acute infection. To evaluate whether IgM or IgG was required, we infected mice deficient in activation-induced cytidine deaminase (AICDA^−/−^), which are unable to undergo class-switch recombination or affinity maturation (29). We also infected mice unable to secrete IgM (sIgM^−/−^) but which can produce membrane-bound IgM and undergo class switch recombination to secrete IgG (30). In contrast to B-cell-deficient μMT mice, survival was not altered in sIgM^−/−^ mice compared to WT mice (Fig. S2a and b). However, male AICDA^−/−^ mice had slightly increased weight loss on day 7 and day 8 PI (Fig. 4a), and 2 of 8 mice succumbed to the infection on day 6 PI (Fig. S2a). This suggests that the early IgG response to CCHFV is required for optimal control of the infection. Interestingly, both male and female sIgM^−/−^ mice had slightly reduced weight loss compared to respective WT mice (Fig. 4a and b), suggesting lack of an early IgM response lessens disease severity. Later in disease, female AICDA^−/−^ mice also had significantly increased weight compared to WT mice (Fig. 4b). We confirmed the expected antibody response in each mouse strain by ELISA, confirming that AICDA^−/−^ and sIgM^−/−^ mice failed to produce CCHFV-specific IgG or IgM, respectively (Fig. S2c and d). Cumulatively, our data indicate that either IgM or IgG is sufficient to control MA-CCHFV infection, although early IgG responses may be necessary for optimal control of the infection.

**Fig 4:**
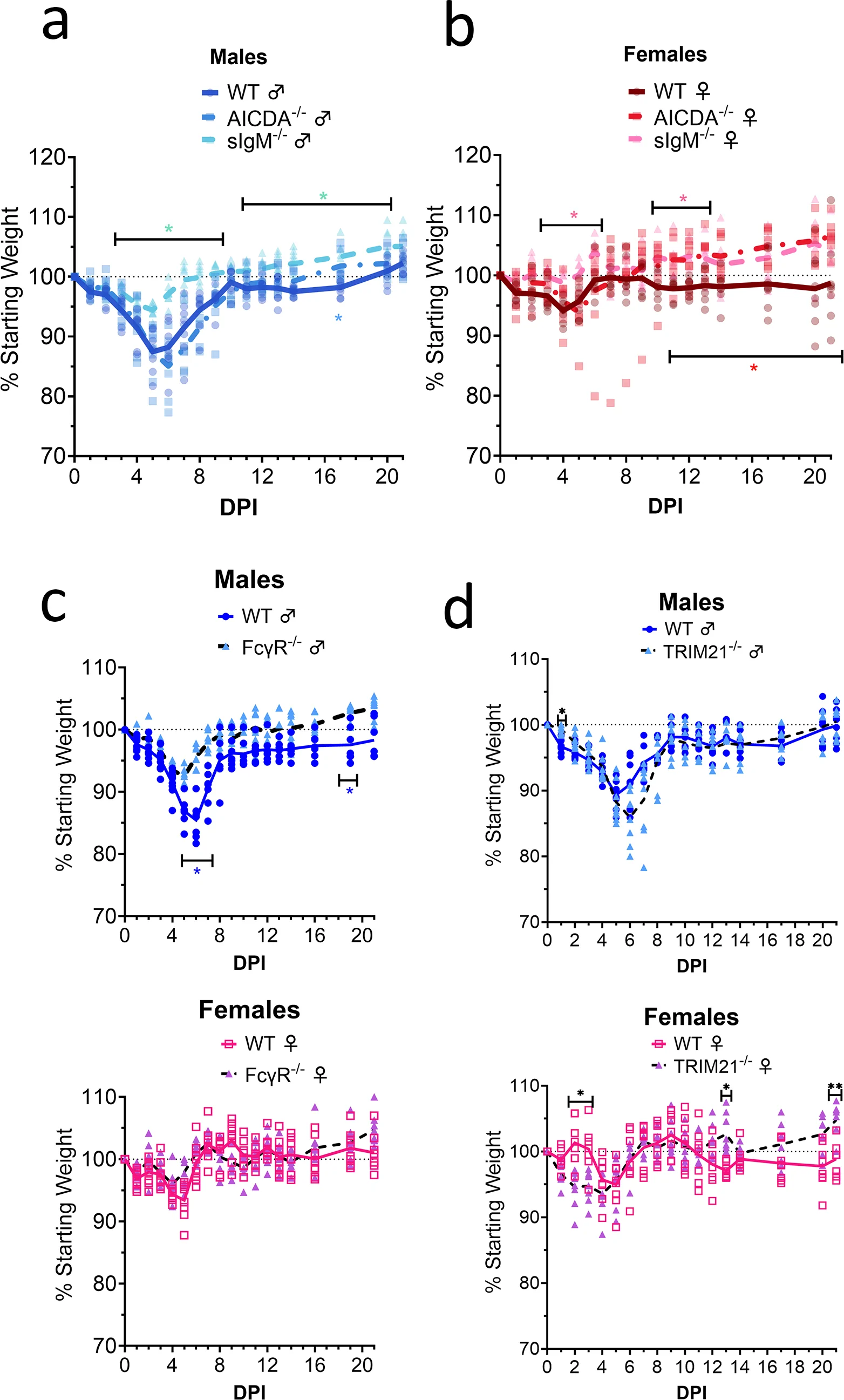
IgM or IgG can control the infection while activating Fc receptors and TRIM21 are dispensable. Male (**a**) or female (**b**) WT, AICDA^−/−^, or sIgM^−/−^ mice were infected with MA-CCHFV and weighed daily. Individual values are shown with a line connecting group means. *P* values were calculated with a repeated-measures mixed-effect model (**a**) or repeated-measures two-way ANOVA (**b**) with Tukey’s multiple comparisons test. WT or FcγR^−/−^ (**c**) or TRIM21^−/−^ (**d**) mice were infected with MA-CCHFV and weighed daily. Individual values are shown with a line connecting group means. *P* values were calculated with a repeated-measures mixed-effect model (male comparisons) or repeated-measures two-way ANOVA (female comparisons) with Sidak’s multiple comparisons test. (**a–d**) *N* = 7–8 per group. *P < 0.05.

### Fc receptors do not contribute to control of MA-CCHFV

Our findings that antibody is required for control of the infection, but neutralizing antibody responses develop after the mice have recovered from acute disease, suggest that Fc-effector functions may be important. We have also recently shown that vaccine-elicited antibodies against the CCHFV NP confer protection through the intracellular Fc receptor tripartite motif containing protein 21 (TRIM21) (31). We infected mice deficient in activating Fc receptors (FcγR^−/−^) or TRIM21 (TRIM21^−/−^) and observed for clinical disease. Surprisingly, male FcγR^−/−^ mice had significantly reduced weight loss on day 6 and day 7 PI compared to WT C57BL6/NTac controls (Fig. 4c). In contrast, female WT and FcγR^−/−^ had similar weight loss. All male and female FcγR^−/−^ mice survived the infection (7/7 and 8/8 respectively), while one male WT mouse out of 8 reached endpoint criteria on day 6 (data not shown). These data suggest that antibody-mediated control of MA-CCHFV does not require activating Fc receptors; instead, absence of these receptors may slightly lessen disease severity.

Infected male TRIM21^−/−^ mice had slightly increased disease with a trend toward increased weight loss, and 1 of 8 mice reached endpoint criteria on day 7 (Fig. 4d and data not shown). Infected female TRIM21^−/−^ had significantly increased weight loss on days 2 and 3 (Fig. 4d). However, at day 3 PI, we were unable to detect a CCHFV-specific IgM or IgG response (Fig. 2a); thus, it is unlikely that the increased weight loss at this time point in female TRIM21^−/−^ mice is due to a CCHFV-specific antibody mechanism. Nevertheless, the mild difference in the phenotype in TRIM21^−/−^ versus WT mice suggests that TRIM21 plays a minimal role in the control of acute MA-CCHFV infection in naïve mice.

### Complement contributes to control of MA-CCHFV infection

We lastly evaluated MA-CCHFV infection in mice deficient in complement activity. Infected male C3^−/−^ mice largely had similar weight loss compared to male WT mice (Fig. 5a). However, survival was significantly worsened in male C3^−/−^ mice, with 45% (9/20) of male C3^−/−^ mice succumbing to the infection (Fig. 5b). Female C3^−/−^ mice had slightly increased weight loss during acute disease (Fig. 5a), although survival was not impacted (Fig. 5b). Consistent with worsened disease and increased mortality, male C3^−/−^ mice had significantly increased viral loads in the blood and liver at day 6 PI (Fig. 5c). Female C3^−/−^ mice had significantly increased viral loads in the blood but significantly reduced viral loads in the spleen at day 6 PI. These data suggest that in both male and female mice, complement contributes to control of the infection in the blood but may have tissue-specific effects in the liver and spleen. Complement has been shown to augment antibody responses (32); thus, it is possible that C3^−/−^ mice had worsened disease due to impaired early antibody responses. We measured antibody responses at day 6 and 21 in WT and C3^−/−^ mice. At day 6 PI, both male and female C3^−/−^ mice had slightly but significantly impaired IgG responses to the infection, but these differences disappeared by day 21 PI (Fig. 5d). Cumulatively, our data indicate that complement contributes to control of the MA-CCHFV infection, while activating Fc receptors and TRIM21 are dispensable.

**Fig 5:**
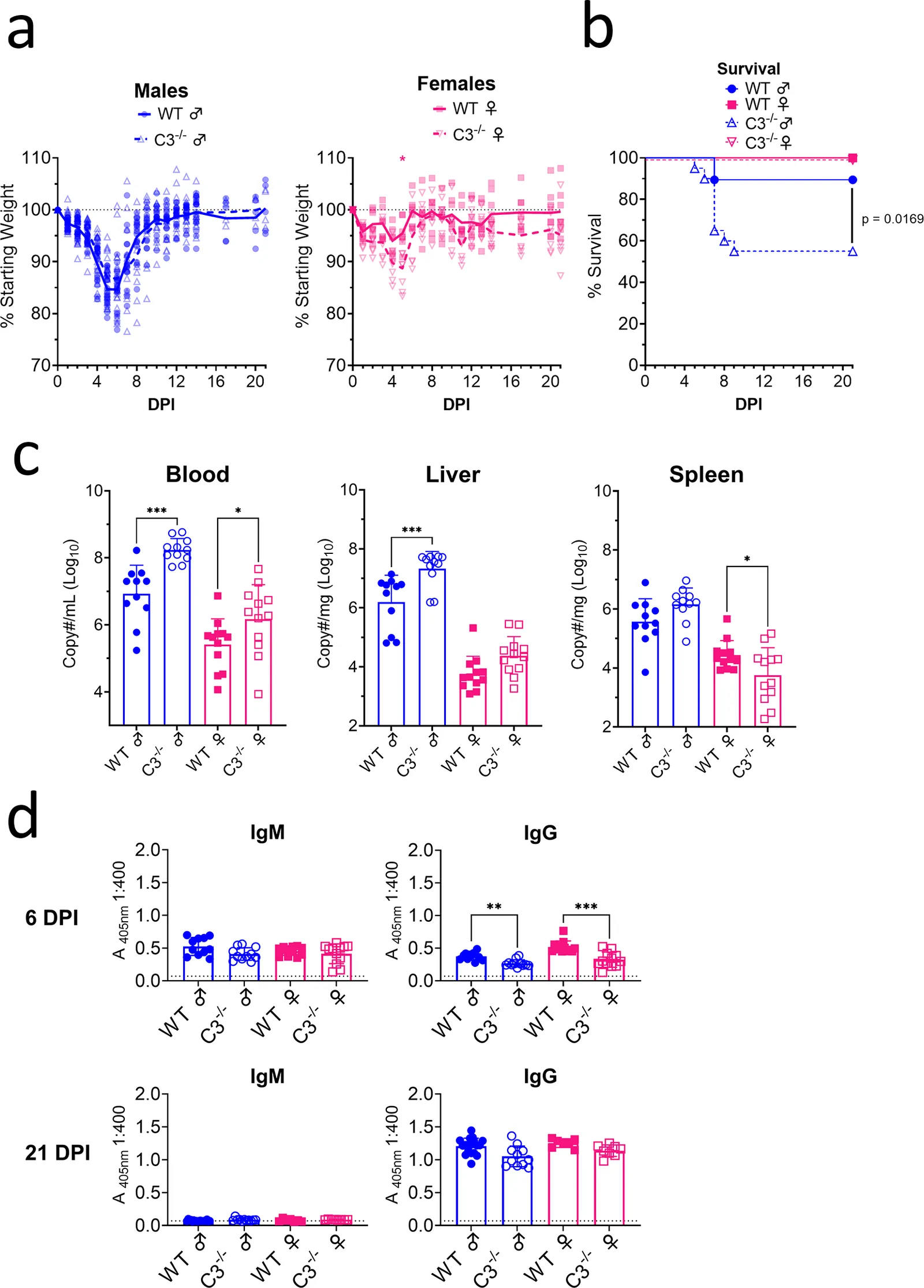
Complement activity is required for control of acute MA-CCHFV infection. Groups of male and female WT or C3^−/−^ mice were infected with MA-CCHFV and weighed daily (**a**) and monitored for survival (**b**). (**a**) *P* values were calculated with a repeated-measures mixed-effect model with Sidak’s multiple comparisons test (males) or two-way ANOVA with Sidak’s multiple comparisons test (females). (**b**) *P* values between respective male and female WT or C3^−/−^ groups were calculated with a log-rank test with Bonferroni’s correction for multiple comparisons. *P* values < 0.025 were considered significant. *N* = 7–20 per group. One male WT mouse was excluded from presentation and analysis due to no apparent clinical signs of disease after challenge. Weight loss for this mouse is included in the raw data. (**c**) Viral RNA in the blood, liver, and spleen measured by qRT-PCR. *P* values for the blood were calculated with a Brown-Forsythe and Welch ANOVA with Dunnett’s multiple comparisons test due to significantly different standard deviations between groups. *P* values for the liver and spleen were calculated with ordinary one-way ANOVA’s with Sidak’s multiple comparisons test. *N* = 11–12 per group. (**d**) CCHFV-specific IgM or IgG antibody was measured at a single dilution by whole-virion ELISA at day 6 and day 21 PI. *P* values were calculated with a Brown-Forsythe and Welch ANOVA with Dunnett’s multiple comparisons test. *N* = 7–18 per group.

## DISCUSSION

Here, we have identified an essential protective role of early humoral immunity in MA-CCHFV-infected mice. We have previously reported that male and female mice exhibit distinct disease upon infection with MA-CCHFV (22) and we are leveraging these sex-linked differences as a model of mild (female) and severe (male) disease to determine the host responses that dictate the disease outcome. Absence of B-cells and antibody led to worsened disease in both male and female mice, suggesting that in both mild and severe CCHFV infections, antibody contributes to control of the infection. However, female mice completely lacking B-cells retain significant control of the infection, exhibiting 100% survival and less weight loss than even WT male mice. Similarly, we reported that female mice depleted of both CD4 and CD8 T-cells retained better control of the infection than WT male mice (23). It is only in female mice completely lacking adaptive immunity (Rag1^−/−^) in which disease was uniformly lethal. Male mice depleted of CD4 and CD8 T-cells exhibited increased disease but no mortality, while lack of humoral immunity resulted in near uniform mortality, suggesting that humoral immunity is more important than cellular immunity for control of the acute infection. Nevertheless, we previously found that depletion of just CD4 T-cells could impair CCHFV-specific antibody responses but did not worsen clinical disease or control of viral replication. In contrast, mice depleted of both CD4 and CD8 T-cells had worsened disease compared to mice depleted of CD8 T-cells alone (23). Together, these findings support a hypothesis that in severe cases of CCHF, cellular immunity can compensate for diminished but not completely absent CCHFV-specific antibody responses. In contrast, during milder disease, either humoral or cellular immunity is sufficient to control the infection.

Our data are consistent with reports that fatal cases of CCHF often have little-to-no antibody responses prior to death but that neutralizing antibody responses may only develop well after resolution of acute disease (13–15). Our data suggest that failure to mount a robust CCHFV-specific antibody response, rather than merely a correlate, may directly lead to poor outcomes in CCHFV-infected humans. The kinetics of the antibody response in MA-CCHFV-infected mice are rapid, with both CCHFV-specific IgM and -IgG detectable by day 6 PI. In CCHFV-infected humans, CCHFV-specific IgG and IgM are similarly rapid with detectable IgM as soon as 2–3 days and IgG within 1 week of symptom onset (14, 15, 33). Our studies in mice deficient in either IgM or IgG suggest that either can contribute to control of the infection. Our data also demonstrate that affinity maturation was not required for control of MA-CCHFV. AICDA^−/−^ mice, in addition to being unable to undergo class switch recombination, also cannot undergo affinity maturation (29) but were, nevertheless, able to control the infection. However, the antibody response to CCHFV continues to mature in WT mice as detectable neutralization activity arose weeks post-infection.

Our data suggest that complement may contribute to control of the infection as C3^−/−^ mice had worsened disease. Complement has a multifaceted role in host responses to viral infections (34), and how it contributes to control of acute CCHFV infection will require further study. Complement has been shown to augment neutralizing and non-neutralizing antibody responses against CCHFV (20, 35), suggesting it may augment the efficacy of CCHFV-specific antibody. Further, our ELISA data demonstrated that C3^−/−^ mice had slightly but significantly impaired early CCHFV-specific IgG antibody responses, suggesting that complement contributes to the rapid CCHFV-specific antibody response. Complement has additional antiviral effector functions such as opsonization of virions, interference of interactions between viral and host proteins, promotion of inflammation, and recruitment of immune cells (36), which could all contribute to restriction of CCHFV. Notably however, C3^−/−^ mice failed to recapitulate the phenotype of mice completely deficient in B-cells. Whereas male μMT mice exhibited near universal lethality upon MA-CCHFV infection, only 45% of male C3^−/−^ mice succumbed to the infection. These data suggest additional antibody effector functions are involved in control of the infection. However, our studies showed that FcγR and TRIM21 are not required. Alternatively, it is possible that multiple Fc-effector mechanisms contribute to control of the infection, and deficiency in a single mechanism is insufficient to recapitulate the phenotype of complete antibody deficiency.

Lastly, our findings showed that the magnitude of the antigen-specific antibody response to acute MA-CCHFV infection was Gc > NP > GP38. This contrasts with findings in CCHFV-infected humans in which the NP is often the dominant target of humoral immunity during acute infection (13) and humans develop protective antibodies against the GP38 (24). These findings likely explain why we recently showed that for vaccine-mediated protection, NP antibodies required TRIM21 but not complement, (31) but here we have shown that TRIM21 was dispensable for control of the acute infection. Interestingly, we have shown that vaccination of the same C57BL6/J mice used here with an RNA-vaccine-expressing parental strain Hoti NP or GPC results in a strong, protective antibody response directed against NP with little-to-no antibody against Gc (16, 31). This demonstrates that C57BL6/J mice can generate antibodies against both NP and Gc and that the context, vaccination versus infection in our studies, can impact antigen-specific antibody responses. MA-CCHFV contains two mutations in the NP that alter two highly conserved amino acids (22), and we cannot exclude that these mutations alter antibody responses against NP. Our commercially sourced recombinant antigens are also based on the prototype CCHFV strain 10200, and it is possible that MA-CCHFV infection elicits strain-specific responses to these antigens. However, as antibodies against the NP or Gc can confer protection against CCHFV (37), further study will be needed to dissect the contribution of antibodies targeting the Gc or NP to protection.

Our study has important limitations. First, although our studies utilized matched wild-type mice on the same C57BL6/J or C57BL6/NTac background as the mutant mice, we cannot exclude the possibility that strain-to-strain variation may account for some of the more subtle phenotypes. Second, although complement-deficient male mice showed increased mortality, they did not fully recapitulate the phenotype of B-cell-deficient mice. This indicates that additional antibody effector functions are required or that multiple effector functions contribute and can partially compensate for the absence of one. Further studies will also be needed to determine what antiviral function of complement restricts CCHFV. Lastly, we failed to detect GP38-specific antibody responses in CCHFV-infected mice. As GP38-specific antibodies can be protective and human CCHFV survivors generate GP38-specific antibodies, our study does not account for the contribution and effector functions required by GP38-specific antibodies in the control of CCHFV during acute human infections.

Cumulatively, we have identified an essential role of CCHFV-specific antibody in the control of the acute infection. Our results suggest that failure to mount an antibody response against CCHFV may directly contribute to poor outcomes. We show that either IgM or IgG can contribute to protection and continue to support the hypothesis that neutralizing antibodies are not required for control of CCHFV. Instead, Fc-effector functions of the early antibody response, such as complement activation, may play a key role. Our previous findings that mice deficient in type I interferon rapidly succumb to MA-CCHFV infection (22) and our recent investigations into how adaptive immunity controls CCHFV support a hypothesis that distinct innate, cellular, and humoral immune responses all contribute to CCHFV morbidity and mortality. Lastly, our findings continue to demonstrate the utility of MA-CCHFV in investigating all aspects of CCHFV pathogenesis in a tractable immunocompetent mouse model.

## MATERIALS AND METHODS

### Animals

C3^−/−^ mice on the C57BL6/J background (stock #029661), AICDA^−/−^ (stock #007770), and wild-type C57BL6/J (stock #00664) mice were purchased and used directly from Jackson Laboratories. TRIM21^−/−^ on the C57BL6/J background (stock #010724) mice were recovered at Jackson Laboratories for an in-house colony established at Rocky Mountain Laboratories, NIAID, NIH. FcγR^−/−^ on the C57BL6/NTac background (Taconic stock #583) were purchased from Taconic, and mice used in studies were from an in-house breeding colony. B6NTac control (stock #B6) mice were purchased and used directly from Taconic Biosciences. sIgM^−/−^ mice were generously provided by Dr. Gudrun Debes, Thomas Jefferson University, and were bred in-house at Rocky Mountain Laboratories. Mice were genotyped to confirm identity prior to use. Mice were humanely euthanized according to the following criteria: ataxia, extreme lethargy (animal is unresponsive to touch), bloody discharge from the nose, mouth, rectum, or urogenital area, tachypnea, dyspnea, or paralysis of the limbs. Although animals were comprehensively evaluated for the above signs, animals that succumbed following MA-CCHFV infection were typically euthanized for extreme lethargy or ataxia.

### Virus and infections

The stock of MA-CCHFV used in these studies was identical to that previously described (22). Virus stocks were deep sequenced to confirm identity and exclude biological contamination. Mice were infected with 10,000 TCID_50_ of MA-CCHFV via a single intraperitoneal injection.

### ELISA

CCHFV-specific IgG or IgM to whole-virus antigen was performed as previously described (38). Briefly, CCHFV strain Hoti was grown in the SW-13 cell culture, the supernatant was collected, clarified of large debris, and the virus was pelleted on a sucrose cushion via ultracentrifugation. Viral pellets were resuspended in phosphate-buffered saline (PBS) with 2% Triton-X 100. The antigen was then applied to high-binding ELISA plates at a 1:1,000 dilution. ELISA against recombinant CCHFV proteins was performed similarly. Recombinant NP, Gc, and GP38 were purchased from The Native Antigen Company and coated onto high-binding ELISA plates at 50 ng of protein per well. Secondary antibodies conjugated to horseradish peroxidase to detect mouse IgG or IgM were purchased from Southern Biotech (#1036-05 or 1021-05, respectively). Plates were developed with two-component ABTS substrate (Seracare). The AUC was calculated using PRISM software with default settings.

### Neutralization assay

Serum neutralization activity against authentic CCHFV Hoti, the parental strain of MA-CCHFV, was measured by microneutralization assay on SW-13 cells, as previously described (16).

### qRT-PCR

RNA from mouse blood was isolated using the QIAamp RNA-mini isolation kit (Qiagen) and from tissues using the RNeasy mini isolation kit (Qiagen), according to the manufacturer’s protocol. Viral RNA was then quantified by qRT-PCR, as previously described (22).

### Statistics

All statistics were done using GraphPad Prism v11.

## Data Availability

The raw data underlying all figures in this article are provided at DOI: 10.6084/m9.figshare.32771790.

## SUPPLEMENTAL MATERIAL

Figure S1 jvi.00987-26-s0001.tif Infection of MD4 transgenic mice with MA-CCHFV.

Figure S2 jvi.00987-26-s0002.tif Survival and antibody responses in MA-CCHFV-infected AICDA^-/-^ and sIgM^-/-^ mice.

ASM does not own the copyrights to Supplemental Material that may be linked to, or accessed through, an article. The authors have granted ASM a non-exclusive, world-wide license to publish the Supplemental Material files. Please contact the corresponding author directly for reuse.

## References

[1] Hawman DW, Feldmann H. 2023. Crimean-Congo haemorrhagic fever virus. Nat Rev Microbiol 21:463–477. doi:10.1038/s41579-023-00871-9 PMC10013989 36918725

[2] Ergönül O. 2006. Crimean-Congo haemorrhagic fever. Lancet Infect Dis 6:203–214. doi:10.1016/S1473-3099(06)70435-2 PMC7185836 16554245

[3] Bente DA, Forrester NL, Watts DM, McAuley AJ, Whitehouse CA, Bray M. 2013. Crimean-Congo hemorrhagic fever: history, epidemiology, pathogenesis, clinical syndrome and genetic diversity. Antiviral Res 100:159–189. doi:10.1016/j.antiviral.2013.07.006 23906741

[4] Johnson S, Henschke N, Maayan N, Mills I, Buckley BS, Kakourou A, Marshall R. 2018. Ribavirin for treating Crimean Congo haemorrhagic fever. Cochrane Database Syst Rev 6:CD012713. doi:10.1002/14651858.CD012713.pub2 PMC5994605 29869797

[5] Spengler JR, Bergeron É, Rollin PE. 2016. Seroepidemiological studies of Crimean-Congo hemorrhagic fever virus in domestic and wild animals. Negl Trop Dis 10:e0004210. doi:10.1371/journal.pntd.0004210 PMC4704823 26741652

[6] Papa A, Bino S, Velo E, Harxhi A, Kota M, Antoniadis A. 2006. Cytokine levels in Crimean-Congo hemorrhagic fever. J Clin Virol 36:272–276. doi:10.1016/j.jcv.2006.04.007 16765637

[7] Papa Anna, Tsergouli K, Çağlayık DY, Bino S, Como N, Uyar Y, Korukluoglu G. 2016. Cytokines as biomarkers of Crimean-Congo hemorrhagic fever. J Med Virol 88:21–27. doi:10.1002/jmv.24312 26118413

[8] Duh D, Saksida A, Petrovec M, Ahmeti S, Dedushaj I, Panning M, Drosten C, Avšič-Županc T. 2007. Viral load as predictor of Crimean-Congo hemorrhagic fever outcome. Emerg Infect Dis 13:1769–1772. doi:10.3201/eid1311.070222 PMC3375790 18217568

[9] Cevik MA, Erbay A, Bodur H, Eren SS, Akinci E, Sener K, Ongürü P, Kubar A. 2007. Viral load as a predictor of outcome in Crimean-Congo hemorrhagic fever. Clin Infect Dis 45:e96–100. doi:10.1086/521244 17806044

[10] Çevik MA, Erbay A, Bodur H, Gülderen E, Baştuğ A, Kubar A, Akıncı E. 2008. Clinical and laboratory features of Crimean-Congo hemorrhagic fever: predictors of fatality. Int J Infect Dis 12:374–379. doi:10.1016/j.ijid.2007.09.010 18063402

[11] Hasanoglu I, Guner R, Carhan A, Kocak Tufan Z, Yagci-Caglayik D, Guven T, Yilmaz GR, Tasyaran MA. 2016. Crucial parameter of the outcome in Crimean Congo hemorrhagic fever: viral load. J Clin Virol 75:42–46. doi:10.1016/j.jcv.2015.12.006 26780111

[12] Ergönül O, Celikbaş A, Dokuzoguz B, Eren S, Baykam N, Esener H. 2004. Characteristics of patients with Crimean-Congo hemorrhagic fever in a recent outbreak in Turkey and impact of oral ribavirin therapy. Clin Infect Dis 39:284–287. doi:10.1086/422000 15307042

[13] Shepherd AJ, Swanepoel R, Leman PA. 1989. Antibody response in Crimean-Congo hemorrhagic fever. Rev Infect Dis 11 Suppl 4:S801–6. doi:10.1093/clinids/11.supplement_4.s801 2501854

[14] Kaya S, Elaldi N, Kubar A, Gursoy N, Yilmaz M, Karakus G, Gunes T, Polat Z, Gozel MG, Engin A, Dokmetas I, Bakir M, Yilmaz N, Sencan M. 2014. Sequential determination of serum viral titers, virus-specific IgG antibodies, and TNF-α, IL-6, IL-10, and IFN-γ levels in patients with Crimean-Congo hemorrhagic fever. BMC Infect Dis 14:416. doi:10.1186/1471-2334-14-416 PMC4133611 25066751

[15] Ergunay K, Kocak Tufan Z, Bulut C, Kinikli S, Demiroz AP, Ozkul A. 2014. Antibody responses and viral load in patients with Crimean-Congo hemorrhagic fever: a comprehensive analysis during the early stages of the infection. Diagn Microbiol Infect Dis 79:31–36. doi:10.1016/j.diagmicrobio.2013.12.015 24630756

[16] Leventhal SS, Meade-White K, Rao D, Haddock E, Leung J, Scott D, Archer J, Randall S, Erasmus JH, Feldmann H, Hawman DW. 2022. Replicating RNA vaccination elicits an unexpected immune response that efficiently protects mice against lethal Crimean-Congo hemorrhagic fever virus challenge. EBioMedicine 82:104188. doi:10.1016/j.ebiom.2022.104188 PMC9335360 35907368

[17] Leventhal SS, Bisom T, Clift D, Rao D, Meade-White K, Shaia C, Murray J, Mihalakakos EA, Hinkley T, Reynolds SJ, Best SM, Erasmus JH, James LC, Feldmann H, Hawman DW. 2024. Antibodies targeting the Crimean-Congo hemorrhagic fever virus nucleoprotein protect via TRIM21. Nat Commun 15:9236. doi:10.1038/s41467-024-53362-7 PMC11511847 39455551

[18] Fels JM, Maurer DP, Herbert AS, Wirchnianski AS, Vergnolle O, Cross RW, Abelson DM, Moyer CL, Mishra AK, Aguilan JT, et al.. 2021. Protective neutralizing antibodies from human survivors of Crimean-Congo hemorrhagic fever. Cell 184:3486–3501. doi:10.1016/j.cell.2021.05.001 PMC8559771 34077751

[19] Mishra AK, Hellert J, Freitas N, Guardado-Calvo P, Haouz A, Fels JM, Maurer DP, Abelson DM, Bornholdt ZA, Walker LM, Chandran K, Cosset F-L, McLellan JS, Rey FA. 2022. Structural basis of synergistic neutralization of Crimean-Congo hemorrhagic fever virus by human antibodies. Science 375:104–109. doi:10.1126/science.abl6502 PMC9771711 34793197

[20] Golden JW, Shoemaker CJ, Lindquist ME, Zeng X, Daye SP, Williams JA, Liu J, Coffin KM, Olschner S, Flusin O, Altamura LA, Kuehl KA, Fitzpatrick CJ, Schmaljohn CS, Garrison AR. 2019. GP38-targeting monoclonal antibodies protect adult mice against lethal Crimean-Congo hemorrhagic fever virus infection. Sci Adv 5:eaaw9535. doi:10.1126/sciadv.aaw9535 PMC6620094 31309159

[5] Pahmeier F, Monticelli SR, Feng X, Hjorth CK, Wang A, Kuehne AI, Bakken RR, Batchelor TG, Lee SE, Middlecamp M, Stuart L, Duarte-Neto AN, Abelson DM, McLellan JS, Biering SB, Herbert AS, Chandran K, Harris E. 2025. Antibodies targeting Crimean-Congo hemorrhagic fever virus GP38 limit vascular leak and viral spread. Sci Transl Med 17. doi:10.1126/scitranslmed.adq5928 PMC12095872 39970234

[22] Hawman DW, Meade-White K, Leventhal S, Feldmann F, Okumura A, Smith B, Scott D, Feldmann H. 2021. Immunocompetent mouse model for Crimean-Congo hemorrhagic fever virus. eLife 10:e63906. doi:10.7554/eLife.63906 PMC7811403 33416494

[23] Rao D, Meade-White K, Leventhal S, Mihalakakos E, Carmody A, Feldmann H, Hawman DW. 2023. CD8+ T-cells target the Crimean-Congo haemorrhagic fever virus Gc protein to control the infection in wild-type mice. EBioMedicine 97:104839. doi:10.1016/j.ebiom.2023.104839 PMC10623175 37866114

[24] Shin OS, Monticelli SR, Hjorth CK, Hornet V, Doyle M, Abelson D, Kuehne AI, Wang A, Bakken RR, Mishra AK, et al.. 2024. Crimean-Congo hemorrhagic fever survivors elicit protective non-neutralizing antibodies that target 11 overlapping regions on glycoprotein GP38. Cell Rep 43:114502. doi:10.1016/j.celrep.2024.114502 PMC11346345 39002130

[25] Bertolotti-Ciarlet A, Smith J, Strecker K, Paragas J, Altamura LA, McFalls JM, Frias-Stäheli N, García-Sastre A, Schmaljohn CS, Doms RW. 2005. Cellular localization and antigenic characterization of Crimean-Congo hemorrhagic fever virus glycoproteins. J Virol 79:6152–6161. doi:10.1128/JVI.79.10.6152-6161.2005 PMC1091677 15858000

[26] Zivcec M, Guerrero LIW, Albariño CG, Bergeron É, Nichol ST, Spiropoulou CF. 2017. Identification of broadly neutralizing monoclonal antibodies against Crimean-Congo hemorrhagic fever virus. Antiviral Res 146:112–120. doi:10.1016/j.antiviral.2017.08.014 PMC7195853 28842265

[27] Homann D, Tishon A, Berger DP, Weigle WO, von Herrath MG, Oldstone MB. 1998. Evidence for an underlying CD4 helper and CD8 T-cell defect in B-cell-deficient mice: failure to clear persistent virus infection after adoptive immunotherapy with virus-specific memory cells from muMT/muMT mice. J Virol 72:9208–9216. doi:10.1128/JVI.72.11.9208-9216.1998 PMC110340 9765468

[28] Goodnow CC, Crosbie J, Adelstein S, Lavoie TB, Smith-Gill SJ, Brink RA, Pritchard-Briscoe H, Wotherspoon JS, Loblay RH, Raphael K. 1988. Altered immunoglobulin expression and functional silencing of self-reactive B lymphocytes in transgenic mice. Nature 334:676–682. doi:10.1038/334676a0 3261841

[29] Muramatsu M, Kinoshita K, Fagarasan S, Yamada S, Shinkai Y, Honjo T. 2000. Class switch recombination and hypermutation require activation-induced cytidine deaminase (AID), a potential RNA editing enzyme. Cell 102:553–563. doi:10.1016/s0092-8674(00)00078-7 11007474

[30] Boes M, Esau C, Fischer MB, Schmidt T, Carroll M, Chen J. 1998. Enhanced B-1 cell development, but impaired IgG antibody responses in mice deficient in secreted IgM. J Immunol 160:4776–4787. doi:10.4049/jimmunol.160.10.4776 9590224

[31] Leventhal SS, Bisom T, Clift D, Rao D, Meade-White K, Shaia C, Murray J, Mihalakakos EA, Hinkley T, Reynolds SJ, Best SM, Erasmus JH, James LC, Feldmann H, Hawman DW. 2024. Antibodies targeting the Crimean-Congo hemorrhagic fever virus nucleoprotein protect via TRIM21. Nat Commun 15:9236. doi:10.1038/s41467-024-53362-7 PMC11511847 39455551

[32] Nielsen CH, Fischer EM, Leslie RG. 2000. The role of complement in the acquired immune response. Immunology 100:4–12. doi:10.1046/j.1365-2567.2000.00009.x PMC2326995 10809953

[33] Burt FJ, Leman PA, Abbott JC, Swanepoel R. 1994. Serodiagnosis of Crimean-Congo haemorrhagic fever. Epidemiol Infect 113:551–562. doi:10.1017/s0950268800068576 PMC2271329 7995364

[34] Stoermer KA, Morrison TE. 2011. Complement and viral pathogenesis. Virology 411:362–373. doi:10.1016/j.virol.2010.12.045 PMC3073741 21292294

[35] Garrison AR, Shoemaker CJ, Golden JW, Fitzpatrick CJ, Suschak JJ, Richards MJ, Badger CV, Six CM, Martin JD, Hannaman D, Zivcec M, Bergeron E, Koehler JW, Schmaljohn CS. 2017. A DNA vaccine for Crimean-Congo hemorrhagic fever protects against disease and death in two lethal mouse models. Negl Trop Dis 11:e0005908. doi:10.1371/journal.pntd.0005908 PMC5619839 28922426

[36] Mellors J, Tipton T, Longet S, Carroll M. 2020. Viral evasion of the complement system and its importance for vaccines and therapeutics. Front Immunol 11:1450. doi:10.3389/fimmu.2020.01450 PMC7363932 32733480

[37] Garrison AR, Moresco V, Zeng X, Cline CR, Ward MD, Ricks KM, Olschner SP, Cazares LH, Karaaslan E, Fitzpatrick CJ, Bergeron É, Pegan SD, Golden JW. 2024. Nucleocapsid protein-specific monoclonal antibodies protect mice against Crimean-Congo hemorrhagic fever virus. Nat Commun 15:1722. doi:10.1038/s41467-024-46110-4 PMC10897337 38409240

[38] Hawman DW, Meade-White K, Haddock E, Habib R, Scott D, Thomas T, Rosenke R, Feldmann H. 2019. Crimean-Congo hemorrhagic fever mouse model recapitulating human convalescence. J Virol 93. doi:10.1128/JVI.00554-19 PMC6714788 31292241

